# Mosquito Rasputin interacts with chikungunya virus nsP3 and determines the infection rate in *Aedes albopictus*

**DOI:** 10.1186/s13071-015-1070-4

**Published:** 2015-09-17

**Authors:** Jelke J. Fros, Corinne Geertsema, Karima Zouache, Jim Baggen, Natalia Domeradzka, Daniël M. van Leeuwen, Jacky Flipse, Just M. Vlak, Anna-Bella Failloux, Gorben P. Pijlman

**Affiliations:** Laboratory of Virology, Wageningen University, Droevendaalsesteeg 1, 6708 PB Wageningen, The Netherlands; Institut Pasteur, Department of Virology, Arboviruses and Insect Vectors, 25-28 rue du Docteur Roux, 75724 Paris, cedex 15, France

**Keywords:** Chikungunya virus, nsP3, Rasputin, Vector competence, *Aedes albopictus*, G3BP

## Abstract

**Background:**

Chikungunya virus (CHIKV) is an arthritogenic alphavirus (family *Togaviridae*), transmitted by *Aedes* species mosquitoes. CHIKV re-emerged in 2004 with multiple outbreaks worldwide and recently reached the Americas where it has infected over a million individuals in a rapidly expanding epidemic. While alphavirus replication is well understood in general, the specific function (s) of non-structural protein nsP3 remain elusive. CHIKV nsP3 modulates the mammalian stress response by preventing stress granule formation through sequestration of G3BP. In mosquitoes, nsP3 is a determinant of vector specificity, but its functional interaction with mosquito proteins is unclear.

**Methods:**

In this research we studied the domains required for localization of CHIKV nsP3 in insect cells and demonstrated its molecular interaction with Rasputin (Rin), the mosquito homologue of G3BP. The biological involvement of Rin in CHIKV infection was investigated in live *Ae. albopictus* mosquitoes.

**Results:**

In insect cells, nsP3 localized as cytoplasmic granules, which was dependent on the central domain and the C-terminal variable region but independent of the N-terminal macrodomain. *Ae. albopictus* Rin displayed a diffuse, cytoplasmic localization, but was effectively sequestered into nsP3-granules upon nsP3 co-expression. Site-directed mutagenesis showed that the Rin-nsP3 interaction involved the NTF2-like domain of Rin and two conserved TFGD repeats in the C-terminal variable domain of nsP3. Although *in vitro* silencing of Rin did not impact nsP3 localization or CHIKV replication in cell culture, Rin depletion *in vivo* significantly decreased the CHIKV infection rate and transmissibility in *Ae.albopictus*.

**Conclusions:**

We identified the nsP3 hypervariable C-terminal domain as a critical factor for granular localization and sequestration of mosquito Rin. Our study offers novel insight into a conserved virus-mosquito interaction at the molecular level, and reveals a strong proviral role for G3BP homologue Rin in live mosquitoes, making the nsP3-Rin interaction a putative target to interfere with the CHIKV transmission cycle.

## Background

Chikungunya virus (CHIKV) is a member of the genus *Alphavirus* (family *Togaviridae*), a group of widely distributed human and animal pathogens. The New world alphaviruses can cause encephalitic disease in humans, while the Old world alphaviruses, including CHIKV, Sindbis virus (SINV), O’nyong nyong virus (ONNV) and Semliki Forest virus (SFV), are associated with rash, fever and (sometimes chronic) arthritis [1]. CHIKV is transmitted by vector mosquitoes and actively replicates in mosquitoes of the genus *Aedes*, in particular *Ae. aegypti* and *Ae. albopictus*. CHIKV is endemic in most of Central Africa and South-East Asia. In 2005-2006, major outbreaks of CHIKV occurred on the Indian Ocean islands of Mayotte, Seychelles, Mauritius and La Réunion, where more than one-third of the population was infected and resultant deaths were reported [1] (Schwartz & Albert, 2010). In 2006-2007 CHIKV caused a major outbreak in India (~1.3 million cases), followed by outbreaks in the rest of South-East Asia [2]. The first autochthonous CHIKV outbreak in Europe occurred in Italy in 2007, where more than 200 people were infected [3]. Likewise, a local CHIKV transmission by *Ae. albopictus* occurred in France in 2010 (2 cases) and 2014 (4 cases) [4, 5]. The outbreak that started in the Caribbean in 2013 has spread to the American main land and by December 2014 over a million cases have been reported throughout the Americas [6, 7]. No licensed vaccine or antiviral treatment against CHIKV is available at present, but many prototype vaccines are in development [8–14].

CHIKV proteins are translated from a viral single-stranded positive-sense RNA of approximately 11.8 kb [15, 16]. The four alphavirus non-structural proteins (nsP1-4) are directly translated from genomic RNA and form a replication complex (RC), which is associated with the plasma membrane and endosomal membranes [17]. A number of functions has been assigned to alphavirus nsPs: nsP1 is involved in capping of RNA [15] and is the membrane anchor of the RC [17], nsP2 has protease and helicase activity, causes host shut-off and inhibits interferon-induced JAK-STAT signaling and the unfolded protein response [18–21]. NsP4 serves as RNA-dependent RNA polymerase [16]. The functions of nsP3 are more enigmatic, but the protein is highly phosphorylated on serine and threonine residues [22, 23] and is essential for RNA synthesis [24] as part of the viral RC [17]. CHIKV nsP3 can be divided into three regions; the macrodomain (amino acids 1-160) is conserved among alphaviruses, *Coronaviridae*, rubella and hepatitis E viruses and can bind ADP-ribose, RNA and DNA *in vitro* [25]*.* The central, zinc-binding domain (amino acids 161-324) is conserved among alphaviruses, while the C-terminal region is highly variable and even shows substantial dissimilarity between CHIKV strains [26, 27].

SINV nsP3 is found in cytoplasmic granules or foci which are also comprised of various host proteins [28, 29]. In both mammalian and mosquito cells the cellular protein Ras-GAP SH3 domain binding proteins (G3BPs) were found in nsP3-granules [28, 29]. G3BPs are ubiquitously expressed proteins conserved among eukaryotes. Mammals have three G3BPs: G3BP1, 2a and 2b, which are expressed from 2 distinct genes, while insects have one, named Rasputin (Rin) [30]. Mammalian G3BP is a widely used marker for stress granules (SGs) [31], which are cytoplasmic messenger ribonucleoproteins (mRNPs) that form when translation is impaired in response to several types of cellular stress [32]. NsP3-G3BP-granules are the explicit phenotype of the first reported function of alphavirus nsP3, as we have recently shown that CHIKV nsP3-G3BP granule formation prevents the establishment of *bona fide* SGs [33]. These nsP3-G3BP-granules did not contain other crucial SG marker eIF3 and cells expressing nsP3 were unable to respond normally to oxidative stress [33]. The inhibition of SGs via an interaction between nsP3 and G3BP has now also been confirmed for SFV [34]. Details on the interaction between nsP3 and mosquito Rin are currently lacking.

Mosquito vectors display different degrees of vector competence for different CHIKV isolates [35]. Vector competence is a complex trait involving an interplay between vectors, pathogens and environmental factors [36] but the molecular details are not well understood. While it has been firmly established that antiviral RNAi pathways play a major role in controlling CHIKV and other arboviral infections in the mosquito [37, 38], other mechanisms of virus-host interactions that influence vector competence and the roles therein of viral (non) structural proteins need to be examined. Recently, however, nsP3 of ONNV (transmitted by *Anopheles* mosquitoes), has been uncovered as an important determinant for vector specificity. CHIKV does not normally infect *An. gambiae,* however, a chimeric virus containing ONNV nsP3 in a CHIKV infectious clone backbone became infectious for *An. gambiae* mosquitoes [39]. Thus, it is hypothesized that specific molecular interactions between mosquito host factors and alphavirus nsP3 determine the vector specificity.

In the present study, we investigated the formation of nsP3-granules in insect cells and elucidated the molecular interactions between nsP3 and Rin. Moreover, we studied the effect of Rin silencing on virus replication in mosquito cell culture and on vector competence for CHIKV in mosquitoes. We show that Rin is an important, proviral determinant for CHIKV infection and dissemination in live mosquitoes.

## Methods

### Cells and viruses

*Spodoptera frugiperda* Sf21 cells were cultured in Grace’s medium (Invitrogen) with 10 % fetal bovine serum (FBS; Invitrogen) and Sf9 cells in Sf900 medium (Invitrogen) with 5 % FBS. *Aedes albopictus* U4.4 cells and C6/36 cells were cultured in Leibovitz’s medium (Invitrogen) supplemented with 10 % FBS, 2 % tryptose phosphate (Invitrogen) and 1 % non-essential amino acids (Invitrogen). All insect cells were cultured at 27 °C. Vero E6 and HEK293t mammalian cells were cultured in Dulbecco’s modified Eagle medium (Invitrogen) supplemented with 10 % FBS at 37 °C and 5 % CO_2_. Infections in cell culture were performed with CHIKV isolate S27 and mosquitoes were infected with CHIKV 06-021 strain.

### Plasmid construction

Cloning of EGFP-nsP3 was described previously [33] and cloned via Gateway technology into pcDNA/Dest40 and pIB-GW plasmid backbones for CMV and OpIE2 driven expression, respectively. Plasmids pIB-EGFP-nsP3.2, pIB-EGFP-nsP3-DDEL, and pIB-EGFP-nsP3-dUGA were generated by PCR from pIB-EGFP-nsP3 using the phosphorylated forward primer attB2-R-phos and reverse primers CHIKVnsP3-2R, CHIKVnsP3-DDEL-R, and CHIKVnsP3-dUGA-R, respectively. Plasmids pIB-EGFP-nsP3.7, pIB-EGFP-nsP3.8, and pIB-EGFP-nsP3.10 were generated from pIB-EGFP-nsP3 and pIB-EGFP-nsP3.2 using the phosphorylated reverse primer EGFP-R-phos and forward primers EcoRI-nsP3-161-F (nsP3.7 and nsP3.8), or EcoRI-nsP3-319-F (nsP3.10). *Ae. albopictus* Rin was amplified by RT-PCR (Invitrogen) from total RNA isolated from U4.4 cells using the Rin forward and reverse primers (Aalb-RIN-F and Aalb-Rin-R) and cloned into pGEM-T easy (Promega) and sequenced (Genbank accession number KP641128). To obtain pIB-Rin-mCherry, Rin was amplified by PCR from pGEM-Teasy-Rin using primers containing *Hin*dIII sites (HindIII-EcoRI-Aalb-Rin-F and HindIII-Aalb-Rin-R) and was inserted as a *Hin*dIII fragment into pIB-mCherry, in frame with and upstream of mCherry. Site-directed mutagenesis of pIB-EGFP-nsP3 and pIB*-*Rin-mCherry was performed using the forward and reverse primers fornsP3-P398A, nsP3-PPR401AAA, nsP3-FG479AA, nsP3-FG497AA, Rin-F34A and Rin-F34W. All constructs were verified by sequencing and primer sequences are listed in Table 1.

**Table 1:** Oligonucleotides used in this study

Name	Sequence
attB2-R-phos	TCATACCCAGCTTTCTTGTAC
CHIKVnsP3-2R	TCAGCGTGATGGCACGTTATGG
CHIKV nsP3-DDEL-R	TCATAATTCGTCGTCCGTGTC
CHIKV nsP3-dUGA-R	TCACCCACCTGCCCTATCTAGTAATTCGTCGTCCGTGTCTG
EGFP-R-phos	CTTGTACAGCTCGTCCATG
EcoRI-nsP3-161-F	CAAGTGGAATTACTAGACGAAC
nsP3-319-F	GTTAGTCCAAGGGAATATAAATC
Aalb-Rin-F	ATGGTAATGGAAGCACAACC
Aalb-Rin-R	CTAACGTCGTCCTCCGTAG
Aalb-Rin-intF	TCCAAGTGTCGCTACC
HindIII-EcoRI-Aalb-Rin-F	GATAAGCTTGAATTCACCATGGTAATGGAAGCACAACC
HindIII-Aalb-Rin-R	GATAAGCTTACGTCGTCCTCCGTAGG
nsP3-FG479AA F	TAACGGCCGCGGATTTTGATGAAGGGGAGA
nsP3-FG479AA R	AATCCGCGGCCGTTATGGGGAAAGTCTCGT
nsP3-FG497AA F	TGACCGCTGCGGACTTCTCGCCGGGCGAAG
nsP3-FG497AA R	GTCCGCAGCGGTCAGTAACTCAGAGGACAA
Rin-F34A F	GCACCGTGCCTACAACAACTCGTCGAGCTTC
Rin-F34A R	TGTTGTAGGCACGGTGCAGGTGATCCGGCG
Rin-F34A F	GCACCGTTGGTACAACAACTCGTCGAGCTTC
Rin-F34A R	TGTTGTACCAACGGTGCAGGTGATCCGGCG
T7-pGEM-Teasy-R	TAATACGACTCACTATAGGGGCCGCGAATTCACTAGTG
Aalb-Rin F2	GTATGCCAACCATTGATCCG
Aalb-Rin R2	GTCCAGTTCGTTCATTGACAG
nsP1-int F	CTGACGGAAGGTAGACGAG
nsP1-int R2	GCACGTGAAGCTGAGCTTCCC
S7 F	CCAGGCTATCCTGGAGTTG
S7 R	GACGTGCTTGCCGGAGAAC
nsP3-P398A F	GAATACCGCGGCAGTCGCA
nsP3-P398A R	TGCGACTGCCGCGGTATTC
nsP3-PPR401AAA F	CGCCAGTCGCAGCGGCCGCAAGAAGACGTGGGAA
nsP3-PPR401AAA R	CCCACGTCTTCTTGCGGCCGCTGCGACTGGCGCGGTAT

### Transient expression of nsP3 and Rin

Insect cells were transfected with the indicated expression plasmids using Fectofly I (Polyplus) or ExpreS^2^ Insect-TR (ExpreS2ion Biotechnologies). Mammalian cells were transfected with lipofectamine 2000 (Invitrogen). Twenty-four hours post transfection the fluorescence of EGFP-nsP3 and/or Rin-mCherry was analysed using a Zeiss Axio Observer Z1m inverted microscope in combination with an X-Cite 120 series lamp.

### Rin Knockdown experiments

Linear DNA of *Ae. albopictus* Rin and firefly luciferase was generated by PCR from pGEM-T easy plasmids using the T7 universal primer (New England Biolabs) and T7-pGEM-Teasy-R and double-stranded (ds) RNA was synthesized *in vitro* with T7 RNA polymerase (Invitrogen). Knockdown in cell culture was performed by transfecting dsRNA into U4.4 cells grown in 24-wells plates (1 μg of RNA per well) using Fugene (promega). One day later, cells were transfected with plasmid pIB-EGFP-nsP3 to monitor nsP3-granule formation or infected with CHIKV at a multiplicity of infection (MOI) of five. At the indicated times post infection, the medium was removed from the cells and used in end point dilution assays on Vero E6 cells. The remaining cells were lysed in TRIzol (life technologies) reagent and total RNA was isolated. The RNA was DNase treated (Applied Biosystems) and reverse transcribed using random primers. Rin, S7 and genomic CHIKV cDNA were amplified (primers: Rin F2/R2, S7 F/R and nsP1 int F/R2) and detected with real-time PCR platinum SYBR Green (Invitrogen), in a Rotor Gene RG-3000 (Corbett Research).

In parallel experiments, cells were washed with PBS and lysed in SDS-loading buffer [100 mM Tris-Cl (pH 6.8), 4 % (w/v) sodium dodecyl sulfate (SDS), 0.2 % (w/v) bromophenol blue, 20 % (v/v) glycerol and 200 mM β-mercaptoethanol]. Samples were heated at 95 °C for 10 min, clarified by centrifugation for one min at 13 000 r.p.m and loaded on a 12 % SDS-Polyacrylamide gel. After electrophoresis, denatured proteins were transferred to an Immobilonmembrane (Millipore) for analysis by Western blotting. Membranes were blocked in 3 % skimmed milk in PBS with 0.05 % Tween 60 (PBST) for 1 h at room temperature. Membranes were washed three times for 5 min each with PBST and subsequently incubated for one hat room temperature with rabbit polyclonal anti-E2 (diluted 1 : 20000; [40]) and anti-β-tubulin (diluted 1 : 4000; Abcam) in PBST, respectively. Membranes were washed and treated with alkaline phosphatase conjugated with goat anti-rabbit IgG mAb (Sigma), diluted 1 : 3000 inPBST, for 45 min at room temperature. Membranes were washed twice for 5 min each with PBST and once for 10 min with AP buffer [100 mM NaCl, 5 mM MgCl2, 100 mM Tris/HCl (pH 9.5), 0.1 % Tween 20]. Proteins were detected by nitro blue tetrazolium chloride/BCIP staining (Roche).

*In vivo* knock down of Rin was performed in *Ae. albopictus* mosquitoes originating from la Reunion island (Providence, F11 generation). 500 ng of dsRin or dsLuc RNA was injected directly in the thorax of female mosquitoes (Drummond nanoject II). Two days post injection mosquitoes were either sacrificed and stored at -80 °C or orally infected with an infectious blood meal containing 10^7^ pfu/ml of CHIKV 06-021 strain. Mosquito rearing and preparation of the infectious blood meal was reported previously [36]. Fully engorged females were selected and incubated in climatic chambers (Binder) at 28 °C, with a light: dark cycle of 16 h: 8 h and 70 % relative humidity. Forced salivation were performed 6 days post-infection as described previously [35]. Saliva and mosquitoes were stored at -80 °C pending further analysis.

### Infectivity assays

Frozen mosquitoes were dissected, separating bodies (abdomen and thorax) from the head. Individual mosquito bodies and heads were homogenized in the bullet blender storm (Next Advance) in 100 μl of DMEM Hepes (Gibco)-buffered medium supplemented with 10 % FBS containing penicillin (100 IU/ml), streptomycin (100 μg/ml), fungizone (2,5 μg/ml) and gentamycin (50 μg/ml) and spun down for 90 s at 14,000 rpm in a table top centrifuge. Thirty μl of the supernatant from the mosquito homogenate or the saliva-containing mixture was incubated on a monolayer of Vero cells in a 96-wells plate. After 2-4 h the medium was replaced by 100 μl of fresh cell culture medium, fully supplemented with antibiotics. Wells were scored for virus specific cytopathic effects (CPE) at three days post infection. Viral titres were determined using 10 μl of the supernatant from the mosquito homogenates in an end point dilution assay on Vero E6 cells. Infections were scored by CPE, three days post infection.

### Statistical analysis

Mosquito bodies and heads were scored positive or negative for CHIKV infection and significant differences were calculated using the Fisher’s exact test (*P* < 0.05). Differences in CHIKV titers (TCID50/ml) in infected mosquito bodies and heads were calculated using the Mann Whitney test (*P* < 0.05).

## Results

### CHIKV nsP3 displays granular localization in both insect and mammalian cells

**Fig. 1:**
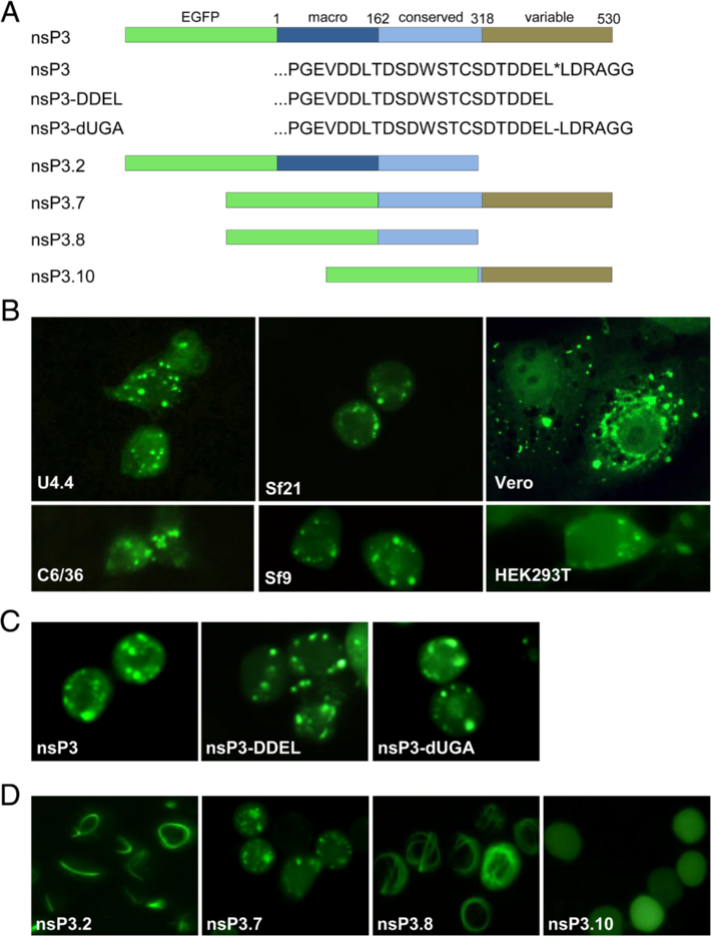
CHIKV nsP3 forms granules in insect cells. **a** Schematic representation of CHIKV nsP3 variants used in this study. Representing either the full length protein N-terminally fused to EGFP (nsP3), nsP3 isoforms with adapted C-terminal amino acids (nsP3, nsP3-DDEL and nsP3-dUAG) or truncated nsP3 variants (nsP3.2, 3.7, 3.8 and 3.10). Asterisk indicates opal stop codon between nsP3 and nsP4. **b** Intracellular distribution of wild type CHIKV nsP3 in cultured insect and mammalian cells. Mosquito and lepidopteran cells were transfected with plasmids that express the nsP3 variants from OpIE2 insect promoters. In mammalian cells expression of the nsP3 variants was driven by a cytomegalovirus promoter. **c** Intracellular distribution of nsP3-DDEL and nsP3-dAUG in insect cells. **d** Intracellular distribution of the truncated nsP3 variants

Previous studies on the alphaviruses SINV, SFV and CHIKV showed that viral nonstructural protein nsP3, either in its authentic form or fused to markers like EGFP or mCherry, localized to cytoplasmic granules in mammalian cells [28, 33, 41, 42]. To investigate if nsP3 has a similar intracellular distribution in insect cells, CHIKV nsP3 was transiently expressed, from an OpIE2 promoter-driven insect expression vector, with a N-terminal EGFP fusion (Fig. 1a) in cell lines derived from mosquitoes (*Ae. albopictus*, U4.4 and C6/36) (Fig. 1b, left) and lepidopteran insects (*Spodoptera frugiperda*, Sf21 and Sf9) (Fig. 1b, middle). As a control, CHIKV nsP3 was also expressed in Vero and HEK293T cells (Fig. 1b, right). In all cell lines tested, CHIKV nsP3 formed cytoplasmic granules (Fig. 1b), which indicates that the intracellular localization of nsP3 is conserved in cells of both vertebrate and invertebrate origin.

The gene encoding CHIKV nsP3 contains a natural leaky (opal) stop codon, six codons upstream of the nsP3-4 cleavage site. Two isoforms of nsP3 are likely to be expressed from this gene during viral infections. To investigate whether both isoforms would have the same intracellular localization, two additional EGFP-fusions were made, one with nsP3 lacking the C-terminal six amino acids (CHIKV nsP3-DDEL) and one with nsP3 lacking the leaky stop codon (CHIKV nsP3-dUGA) (Fig. 1a). When transiently expressed in insect cells, CHIKV nsP3 and the two isoforms displayed an identical granular localization (Fig. 1c), which shows that the terminal six amino acids of CHIKV nsP3 do not impact its subcellular localization.

### The conserved domain of CHIKV nsP3 is sufficient for multimerization but the variable domain is required for the formation of nsP3-granules

In mammalian cells, the C-terminal variable domain was found to be essential for nsP3 granule formation, and upon deletion the localization changed to a filamentous phenotype [33]. To determine which domains within nsP3 are responsible for the formation of nsP3-granules, truncated versions of nsP3 fused with EGFP (Fig. 1a) were expressed in insect cells (Fig. 1d). For these studies we used Sf21 cells because of their superior transfection efficiency as compared to mosquito cells. Removal of the entire C-terminal variable region (nsP3.2) resulted in the formation of filamentous, cytoplasmic structures. (Fig. 1d, left). To investigate whether the macrodomain could be eliminated from nsP3 without affecting its localization, it was deleted from EGFP-fused nsP3 and nsP3.2, yielding truncated mutants nsP3.7 and nsP3.8, respectively (Fig. 1a). When expressed in insect cells, nsP3.7 showed an identical granular phenotype as full-length nsP3, whereas nsP3.8 formed filaments that were very similar to those produced by nsP3.2 (Fig. 1d, middle). We observed similar filaments upon expression of these nsP3.2 or nsP3.8 constructs in mammalian cells, and showed that they did not colocalize with cytoskeleton markers actin or tubulin [33]. To investigate if the C-terminal, variable region alone could cause granule formation, it was N-terminally fused to EGFP (nsP3.10) (Fig. 1a) and expressed in insect cells. The localization of nsP3.10 was diffuse, nuclear-cytoplasmic (Fig. 1d, right), showing that the C-terminal region of CHIKV nsP3 is required, but not sufficient for the formation of nsP3 granules.

### NsP3 granules co-localize with Rasputin, the insect homolog of mammalian G3BP

**Fig. 2:**
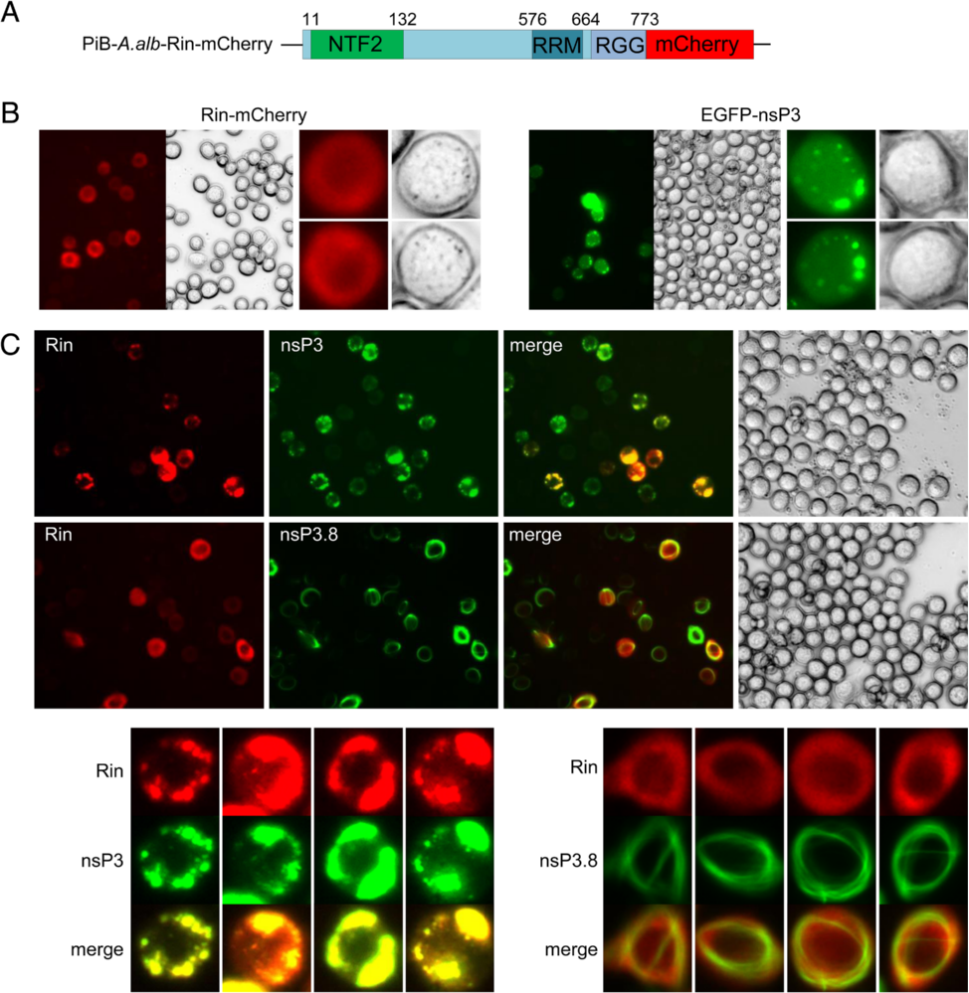
CHIKV nsP3 sequesters mosquito Rasputin into cytoplasmic granules. **a** Schematic representation of *Ae.albopictus*-Rin-mCherry. NTF2; nuclear transport factor 2-like domain, RRM: RNA recognition motif, RGG; arginine-glycine rich box. **b** *Ae.albopictus*-Rin-mCherry or EGFP-nsP3 were transiently expressed in SF21 insect cells and display diffuse and granular intracellular distributions, respectively. **c** *Ae.albopictus*-Rin-mCherry and EGFP-nsP3 (top) or EGFP-nsP3.8 *(bottom)* were co-transfected into insect cells. Magnified images are presented

In SINV nsP3 pull-down studies, G3BP and its insect homologue Rasputin (Rin) were identified as predominant nsP3-interacting proteins in virus-infected mammalian and mosquito cells, respectively [28]. To study a putative interaction of CHIKV nsP3 with mosquito Rin in live cells, the gene encoding *Ae. albopictus* Rin was amplified by RT-PCR from total RNA isolated from U4.4 cells using PCR primers specific for *Ae. aegypti* Rin. We cloned and sequenced the obtained amplicon. The *Ae. albopictus* Rin sequence (Genbank:KP641128) was 307 amino acids longer than human G3BP1, but the nuclear transport factor 2 (NTF2)-like domain, the RNA recognition motif (RRM) and the arginine glycine-rich (RGG-) box were conserved between these species (Fig. 2a).

The subcellular localization of Rin was studied by transient expression in insect cells as C-terminal fusion with mCherry, in a similar fashion to a previously described and functional G3BP-EGFP fusion [31]. When expressed in insect cells, Rin was evenly distributed throughout the cytoplasm (Fig.2b, left). However, when Rin was co-expressed with nsP3, which localized to small nsP3 granules (Fig. 2b, right), both proteins displayed strong co-localization and formed much larger granules (Fig. 2c, top). These nsP3- and Rin-positive granules or aggregates were larger and more asymmetrical than normal nsP3-granules. In this experiment, Rin was transiently (over) expressed, which may explain the large size of the granules. In contrast, when mCherry-Rin was co-expressed with the C-terminal truncated, filamentous mutants EGFP-nsP3.8 (Fig. 2c, bottom) or EGF-nsP3.2 (not shown), Rin did not co-localize with the filaments formed by these mutants and retained its diffuse, cytoplasmic localization (Fig. 2c, bottom). In conclusion, the C-terminal hypervariable domain of nsP3 is important for the interaction with Rin in insect cells.

### The C-terminal TFGD repeats in the variable domain of CHIKV nsP3 interact with Rasputin

**Fig. 3:**
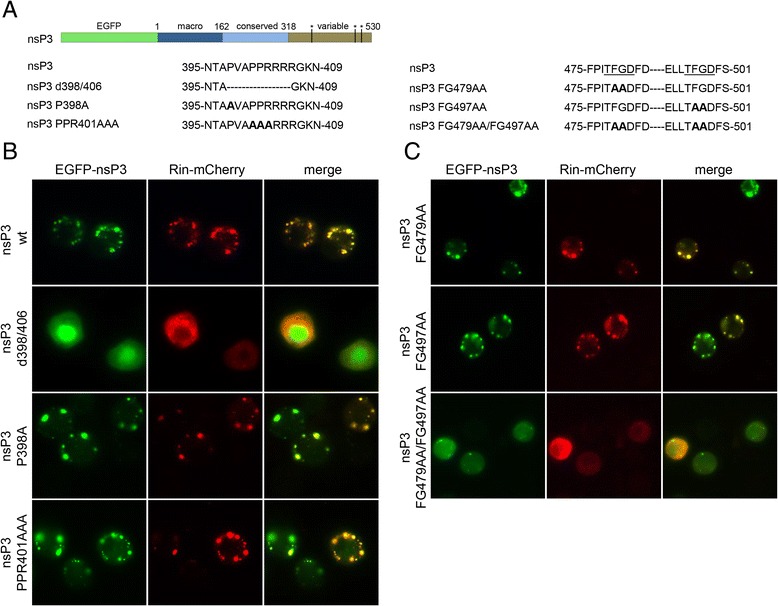
The C-terminal TFGD repeats of nsP3 interact with mosquito Rasputin. **a** Schematic representation of nsP3 and the conserved domains within the C-terminal variable domain. Deletions and mutations are indicated in the amino acid sequence. Insect cells (Sf21) were co-transfected with Rin-mCherry and either one of the EGFP-nsP3 variants. **b** SH3-domain binding motif, nsP3-d398/406, nsP3-P398A or nsP3-PPR401AAA. **c** C-terminal repeats, EGFP-nsP3-FG479AA, EGFP-nsP3-FG497AA or EGFP-nsP3-FG479AA/FG497AA

Previously we showed that transiently expressed CHIKV nsP3 sequesters G3BP into cytoplasmic granules in mammalian cells. Deletion of a conserved SH3-domain binding motif (PVAPPRRRR) in the variable domain of nsP3 resulted in a diffuse nuclear/cytoplasmic localization of nsP3 and abrogated the interaction between nsP3 and G3BP, restoring the potential of the cell to respond to oxidative stress [33]. A recent study, however, convincingly showed that the interaction between nsP3 and mammalian G3BP depends on two conserved repeats (with core sequence TFGD) in the variable domain of nsP3 [43]. To further investigate which domain(s) of nsP3 are crucial for the interaction between CHIKV nsP3 and Rin, both the SH3-domain binding motif and conserved TFGD repeats in the variable domain of nsP3 were mutated using site-directed mutagenesis (Fig. 3a). When EGFP-nsP3 and Rin-mCherry were transiently expressed in insect cells, both wild type proteins displayed perfect co-localization. Deletion of the SH3-domain binding motif (nsP3-d398/406) resulted in a diffuse nuclear/cytoplasmic distribution of nsP3 and a diffuse mainly cytoplasmic localization of Rin (Fig. 3b, second panel), identical to the distribution of nsP3 and G3BP in mammalian cells [33]. However, when conserved proline and/or argenine residues from the SH3-domain binding motif were substituted for alanines (nsP3-P398A and nsP3-PPR401AAA) the co-localization of nsP3 and Rin in cytoplasmic nsP3-granules remained unchanged (Fig. 3b).

Next, we mutated both domains of the two conserved TFGD repeats separately or together resulting in pIB-EGFP-nsP3-FG479AA, pIB-EGFP-nsP3-FG497AA, and pIB-EGFP-nsP3-FG479AA/FG497AA. The single and double EGFP-nsP3 TFGD mutants were transiently expressed in insect cells together with Rin-mCherry. Both the single FG479AA and FG497AA mutants still sequestered Rin into nsP3-granules (Fig. 3c, top and middle panels). The double TFGD mutant, however, displayed a completely diffuse intracellular distribution of Rin (Fig. 3c, bottom panel) but retained a normal granular distribution similar to wildtype EGFP-nsP3 (Fig. 1b).

These results indicate that deletion of the SH3-domain binding motif does abrogate the formation of nsP3 granules, but the formation of nsP3-granules and the interaction with Rin is retained when conserved amino acids within this domain are substituted for alanines. However, the formation of CHIKV nsP3-Rin-granules is also abrogated when both the C-terminal conserved TFGD repeats are mutated, suggesting that these motifs are involved in the nsP3-Rin interaction.

### The NTF2-like domain of mosquito Rasputin interacts with CHIKV nsP3

**Fig. 4:**
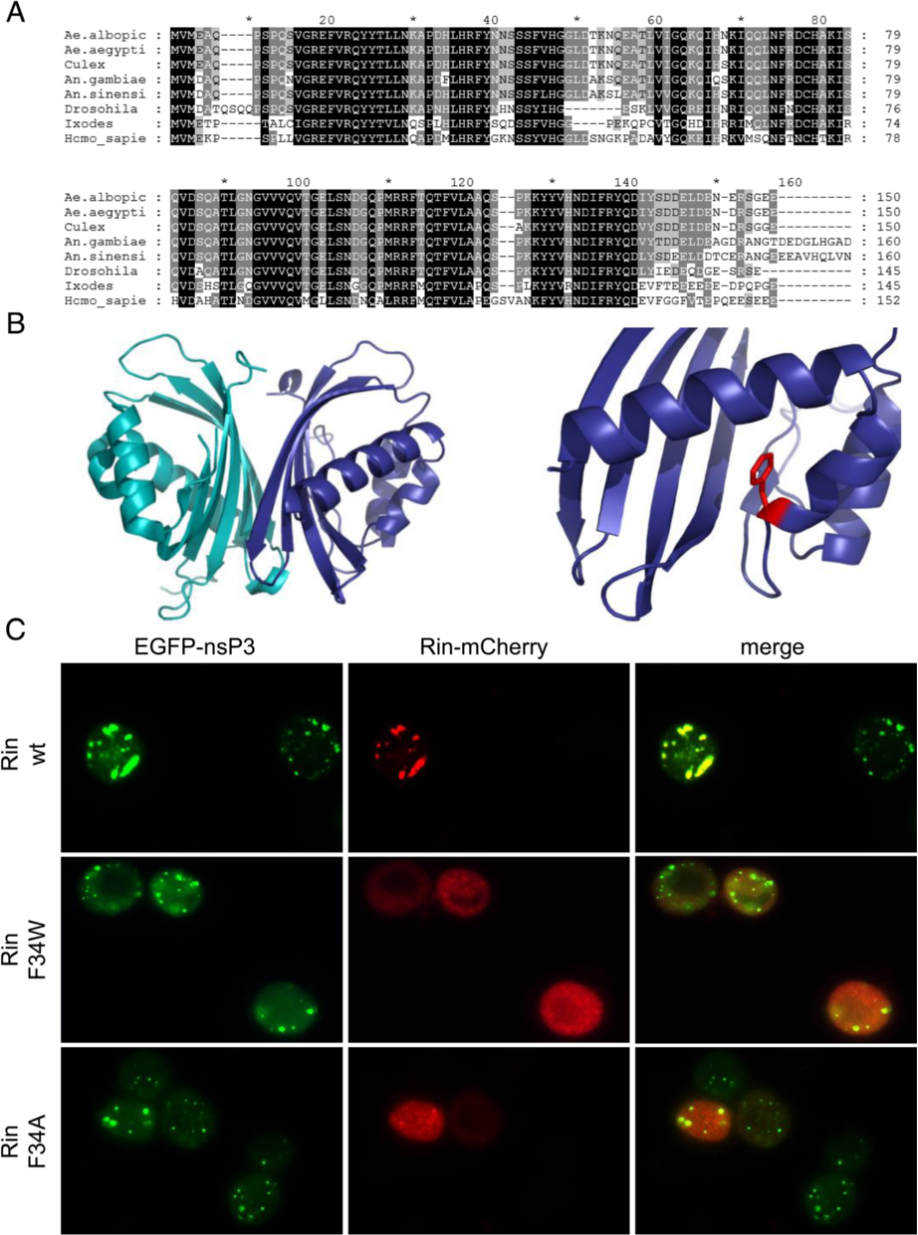
nsP3 interacts with the NTF2-like domain of mosquito Rasputin. **a** Protein alignment of the Rin/G3BP NTF2-like domains from *Ae. albopictus* (KP641128), *Ae. aegypti*, (XP_001651045), *Culex quincefasciatus* (XP_001861860), *Anopheles gambiae* (XP_001688309), *Anopheles sinensis* (KFB40464), *Drosophila melanogaster* (AF231031), *Ixodes ricinus* (GANP01009274), and *Homo sapiens* (CAG38772). Genbank accession numbers in brackets. Alignment made with CLUSTALX and modified using Genedoc. **b** Structural modeling of *Ae. albopictus*-Rin modeled onto *D. melanogaster* Rin. Modeling was performed using the Phyre2 server (www.sbg.bio.ic.ac.uk/phyre2), results were visualized with PyMOL (www.pymol.org). Left, *Ae. albopictus* Rin (deep blue) is depicted together with *D. melanogaster* Rin (cyan). Right, the FxFG binding pocket is shown and phenylalanine 34 is highlighted in red. **c** Sf21 insect cells were co-transfected with EGFP-nsP3 and either Rin-mCherry (wild type), Rin-mCherry F34A or Rin-mCherry F34W

The NTF2-like domain of G3BP has been shown to interact with SINV nsP3 in mammalian cells [29]. The first 140 amino acids of mosquito Rin show high sequence homology to that of NTF2-like domains including the NTF2-like domain of human G3BP (Fig. 4a). Protein structure prediction of *Ae. albopictus* Rin revealed a 100 % structural homology of the NTF2-like domain with those of G3BP and *Drosophila* Rin (Fig. 4b, left), which contains a binding pocket for FxFG containing peptides [44, 45]. Here we mutated a phenylalanine of Rin (F34) that is expected to interact with the phenylalanines of FxFG domains via pi-stacking (Fig. 4b, right) [44]. Indeed, amino acid substitutions in Rin (F34W or F34A), within PiB-A.alb-Rin-mCherry (Fig. 2a) strongly reduced the sequestration of Rin into nsP3-granules (Fig. 4c), indicating that this amino acid is essential for nsP3-Rin interaction.

### Effect of Rasputin silencing on the formation of CHIKV nsP3-granules

**Fig. 5:**
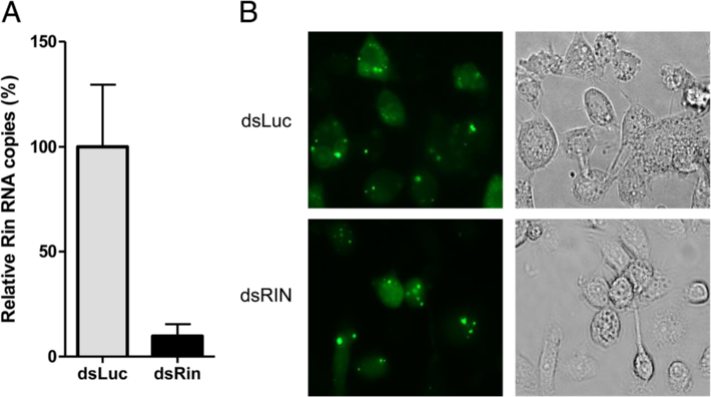
Formation of nsP3-granules is independent of mosquito Rasputin. **a**. Rin silencing was determined by semi-quantitative RT-PCR on *Ae. albopictus* Rin mRNA, relative for the internal control S7. Bars represent relative Rin mRNA expression normalized to dsLuc transfected samples. Error bars indicate standard deviation of duplicate samples from a single representative experiment, which is presented in B. **b**. U4.4 mosquito cells were transfected with dsRNA against *Ae. albopictus* Rin or luciferase. Twenty-four hours later these cells were transfected with wild type EGFP-nsP3, displaying a granular localization

So far, we have shown that nsP3 has a granular localization and that Rin has a diffuse cytoplasmic localization when expressed individually. To investigate whether the formation of nsP3-granules requires Rin, localization of EGFP-nsP3 was studied after Rin expression was silenced in mosquito cells using dsRNA-mediated RNAi. U4.4 mosquito cells were transfected with dsRNA from either Rin (dsRin) or firefly luciferase (dsLuc) as a control. Semi-quantitative RT-PCR on *Ae. albopictus* Rin mRNA, normalized by housekeeping gene S7 mRNA, showed a 90 % reduction in Rin mRNA when cells were transfected with dsRin RNA (Fig. 5a). Subsequent transient expression of EGFP-nsP3 displayed clear nsP3-granules in both dsRin and dsLuc transfected cells (Fig. 5b). This result indicates that the formation of CHIKV nsP3-granules in mosquito cells is independent of (high levels of) mosquito Rasputin.

### Rasputin silencing reduces the CHIKV infection rate in *Aedes albopictus* without affecting CHIKV infection in cell culture

**Fig. 6:**
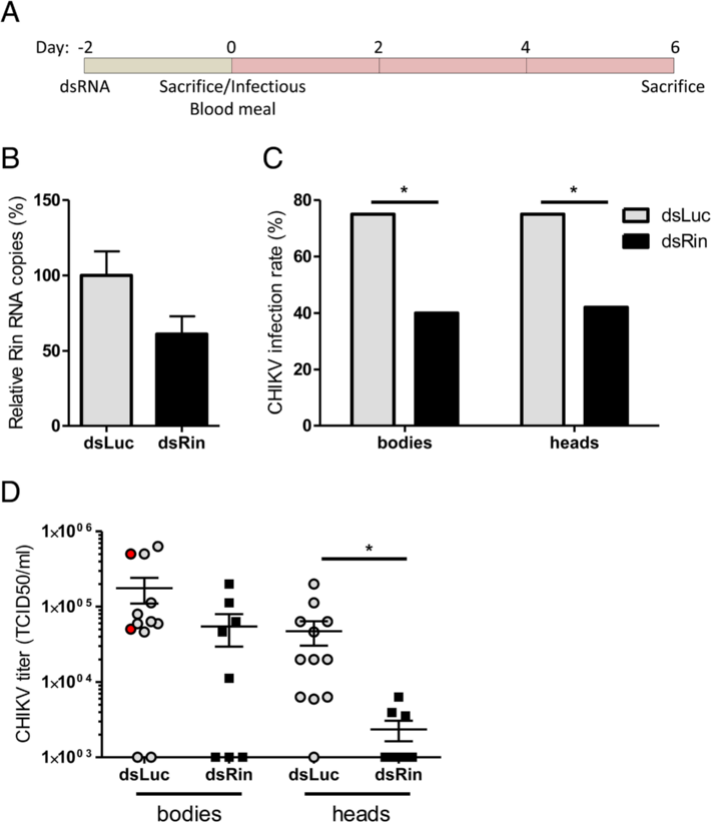
*In vivo* Rin silencing reduces the CHIKV infection rate in *Aedes albopictus* mosquitoes. **a** Schematic representation of the experiment. Mosquitoes were injected with 500 ng of dsRin or dsLuc two days prior to blood feeding. On day 0, a subset of mosquitoes was sacrificed to determine the level of Rin depletion. Remaining mosquitoes were orally infected with CHIKV (10^7^pfu/ml) and sacrificed six days post infection. **b** Total RNA was isolated and Rin silencing was determined by semi-quantitative RT-PCR on *Ae. albopicutus* Rin mRNA, normalized for the internal control S7. Bars represent mean Rin mRNA values normalized to dsLuc injected mosquitoes. Error bars indicate one standard error of the mean (*n* = 5). **c** Heads and bodies from the blood fed mosquitoes were separated, homogenized and the presence of CHIKV was determined by incubating the homogenate on Vero E6 cells. Bars represent the percentage of CHIKV positive mosquito bodies and heads from both dsLuc and dsRin injected mosquitoes. Asterisk indicates significant difference (*P* < 0.05 Fisher’s exact test). **d** From all the CHIKV positive mosquito heads and bodies the viral titers (TCID^50^/ml) were determined. Data points represent one individual mosquito head or body. Asterisk indicates significant difference between dsLuc and dsRin injected mosquitoes (*P* < 0.05, Mann Whitney test) and red data points indicate mosquitoes with positive saliva

As alphavirus nsP3 is an important determinant for vector specificity [39] and specifically interacts with Rin, we investigated the putative role for Rin during CHIKV infection in live mosquitoes. *Ae. albopictus* females (5-day old, 200 per group) were intrathoracically injected with 500 ng dsRNA against Rin or Fluc (day -2). Two days later (day 0), the mosquitoes were offered a blood meal containing 10^7^ pfu/ml CHIKV. Six days after blood feeding saliva was isolated and the mosquitoes were sacrificed (Day 6) (Fig. 6a). Silencing of Rin mRNA was confirmed in mosquitoes on the day of the blood meal, two days post dsRNA injections. Total RNA was isolated and relative Rin mRNA copies were quantified. Mean values in relative Rin mRNA copies in the dsRin injected mosquitoes were 60 % of the dsLuc injected mosquitoes (Fig. 6b).

On day six post infection, mosquito saliva was obtained and later the mosquito heads were separated from their thorax and abdomen, to be able to distinguish between infections that were transmissible, fully disseminated or limited to the mosquito body, respectively. Mosquito saliva or homogenate from either heads or bodies were incubated on Vero cells to determine the presence of CHIKV. CHIKV infected 75 % of the mosquitoes that were injected with dsLuc RNA (Fig. 6c). In these mosquitoes the infection had also disseminated into the head of the mosquito. In addition, it resulted in two mosquitoes with infectious saliva (Fig. 6d, red symbols). Injection with dsRin significantly reduced (*P* < 0.05) the number of infected mosquito bodies and heads to 40 % (Fig. 6c). No mosquitoes had infectious saliva. Furthermore, Rin silencing also reduced the viral titers in the infected mosquitoes, with a significant >20-fold reduction in the mosquito heads (*P* < 0.05, Fig. 6d). Together, these results show a significant effect of Rin silencing on the infection rate and dissemination of CHIKV in *Ae. albopictus*.

**Fig. 7:**
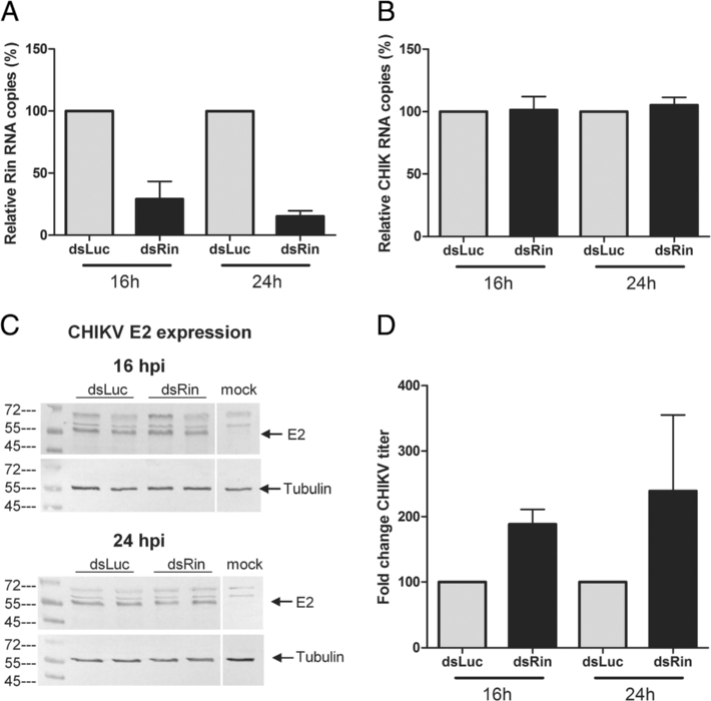
CHIKV infection *in vitro* is not affected by Rin depletion. In four independent experiments, U4.4 mosquito cells were transfected with dsRNA against *Ae. albopictus* Rin or luciferase. Twenty-four hours later these cells were infected with CHIKV (MOI 5) and total RNA was isolated at 16 or 24 hpi. **a** Rin silencing was determined by semi-quantitative RT-PCR on *Ae. albopictus* Rin mRNA, normalized for the internal control S7. **b** CHIKV genomic RNA was quantified with primers that anneal to the nsP1 gene. **c** CHIKV structural protein expression was determined by immunoblot staining against CHIKV E2 and host cell β-tubulin. Protein sizes indicated in kDa. Results of duplicate experiments are shown. **d** At the indicated hpi medium was harvested and the CHIKV titer (TCID_50_/ml) was determined in end point dilution essays. A,B,D. Bars represent the mean of four independent experiments, which have been normalized to the respective value of dsLuc transfected samples in each individual experiment. Error bars represent one standard error of the mean

To establish whether or not the strong negative effect of Rin silencing on the infection rate and dissemination observed *in vivo* could simply be explained by an overall reduced efficiency of virus replication, we examined the effects of Rin silencing *in vitro* on CHIKV infection in mosquito cell culture. U4.4 mosquito cells were transfected with dsRin or dsLuc. Twenty four hours later, cells were infected with CHIKV at an MOI of five. Sixteen and twenty-four hpi, total RNA was isolated, viral structural proteins were detected and viral titers were determined. Semi-quantitative RT-PCR on transcripts purified from CHIKV infected cells showed that Rin silencing was efficient (>70 %) (Fig. 7a). To quantify replication levels of CHIKV in Rin silenced cells the relative levels of CHIKV genomic RNA were measured. CHIKV replication produced equal concentrations of genomic RNA in Rin-depleted versus dsLuc transfected cells at both timepoints (Fig. 7b). Structural protein expression of CHIKV was detected in an immunoblot using E2 polyclonal antiserum, with no apparent differences between dsRin and dsLuc transfected cells, at both timepoints (Fig. 7c, arrow). Correspondingly, the CHIKV titer in Rin-depleted cells was similar to the titer in cells transfected with dsLuc RNA (Fig. 7d). We conclude that Rin depletion in cultured U4.4 mosquito cells does not affect CHIKV RNA replication, structural protein expression or virion production. This result suggests that Rin is an important host factor required for CHIKV infection and dissemination *in vivo* without directly affecting CHIKV replication kinetics in *in vitro* cell culture.

## Discussion and conclusions

In this study we investigated the localization of CHIKV nsP3 and its interaction with mosquito Rin in insect cells and live mosquitoes. Our results show that the intracellular distribution of CHIKV nsP3 is conserved in cells from mammalian and insect origin. In mosquito cells, CHIKV nsP3 forms cytoplasmic granules, which are highly similar to the nsP3-G3BP granules that inhibit the formation of SGs in mammalian cells [33]. Removal of the variable domain results in the formation of filaments. Both the granular or filamentous nsP3 structures form independent of the N-terminal macrodomain. This indicates that multimerization of nsP3 is attributed to the central conserved domain. How nsP3 multimerizes into these two diverse cytoplasmic phenotypes is unknown, however, removal of the C-terminal variable domain may cause a conformational change or affect interactions with host factors which allows nsP3 to form long cytoplasmic filaments. Whereas Rin is clearly sequestered into nsP3-granules and transient overexpression of Rin may increase the size of the nsP3-granules, silencing of Rin and reducing its co-localization with nsP3 by mutagenesis shows that Rin is not required for the formation of nsP3-granules (Figs. 3, 4 and 5). Elucidation of the exact structural composition of these nsP3 granules and filaments, however, needs further experimentation.

In mammalian cells, nsP3-granule formation and the inhibition of SGs is lost when the conserved SH3-domain binding motif is removed from the variable domain of nsP3 [33]. Similarly, nsP3-d398/406 was diffuse throughout insect cells. Amino acid substitutions within the SH3-domain binding motif, however, did not affect the formation of nsP3-granules or the sequestration of mosquito Rin into granules. Apparently, the amino acid substitutions were not sufficient to abrogate the interaction between nsP3 and Rin. Alternatively, deletion of the complete SH3-domain binding motif may have disrupted the folding of nsP3, rendering a dysfunctional protein that can no longer execute its normal function. Indeed, deletion of the entire SH3-domain binding motif from a CHIKV replicon yielded a replication-negative phenotype [33].

G3BP and nsP3 were also shown to interact via two conserved repeats in the C-terminal variable domain of nsP3 [43]. When we replaced the phenylalanine and glycine from either one of the nsP3 C-terminal TFGD repeat with alanines there was no apparent change in the co-localization of nsP3 and Rin. However, the interaction between nsP3 and Rin was completely lost when both TFGD repeats were mutated (Fig. 3c). This suggests a direct interaction between these amino acid repeats and Rin, and shows that both repeats are redundant for the interaction with Rin. *Ae. albopictus* Rin was isolated from U4.4 cells. Sequence analysis revealed that the N-terminal NTF2-like domain has high homology with other NTF2-like domains including human G3BP (Fig. 4a). The three-dimensional crystal structures of the NTF2-like domains from *Drosophila* Rin and human G3BP have recently been resolved, and contain a binding pocket for FxFG containing peptides [44, 45]. The NTF2-like domain from *Ae. albopictus* Rin was modelled onto that of *Drosophila*, showing high resemblance (Fig. 4b). As expected from this model, point mutations in the binding pocket of the Rin NTF2-like domain (position F34) greatly reduced the interaction between nsP3 and Rin (Fig. 4c). Although Rin still partly localized to nsP3-granules, this result does provide evidence of an interaction between CHIKV nsP3 and the NTF2-like FxFG binding pocket of Rin. A recent study has confirmed this interaction between homologous sites in SFV nsP3 and mammalian G3BP [46]. Additional interactions were predicted between FxFG peptides and residues in the NTF2-like binding pocket of G3BP [44], which could explain the strongly reduced, but not completely abolished, interaction of mutated Rin with nsP3.

Rasputin silencing during oral, *in vivo* infections resulted in a marked decrease in the percentage of CHIKV infected mosquitoes in concert with strongly reduced viral titers in the mosquito heads (Fig. 6). Interestingly, *in vitro* Rin silencing did not affect CHIKV infection in cultured mosquito cells (Fig. 7), which is in agreement with *in vitro* studies with SINV and siRNA-mediated G3BP1/2 silencing in mammalian cells [47]. However, simultaneous G3BP1/2 silencing reduced early CHIKV replication in 293/ACE2 cells [48]. These observations suggest that Rin may be involved in the initial establishment of a productive infection and/or affects CHIKV infections in specific mosquito tissues, e.g. the midgut. It also is an indication that results obtained in cell lines are not always a good proxy for results obtained *in vivo*. Indeed, midgut barriers have been described in arthropods that limit arbovirus replication and/or dissemination through the organism [49, 50]. The interaction between nsP3 and Rin may play a significant role in modulating the midgut antiviral responses. Interestingly, exchanging the nsP3 genes of CHIKV and ONNV made CHIKV infectious for *An. gambiae* [39]*.* Moreover, replacing only the C-terminal end of CHIKV nsP3, which is required for Rin interaction, with that of ONNV was sufficient to orally infect *An. gambiae* with CHIKV. This fragment encompasses the variable domain of CHIK nsP3, suggesting a strong role for the C-terminal domain of nsP3 in facilitating oral infection in specific vector species.

The decreased infectivity of CHIKV in Rin depleted mosquitoes suggests a proviral role for Rin. In *Drosophila*, Rin is involved in Ras and Rho-mediated signaling, cell proliferation and oogenesis and has been suggested to form RNase inhibitor complexes [51–53], which could protect CHIKV RNA replication during the initial infection in the mosquito midgut. Clearly, the molecular details of the nsP3-Rin interaction in mosquitoes should be examined in follow up studies to uncover the exact mechanism.

In mammalian cells, G3BP is essential for assembly of SGs, which display many antiviral characteristics [54, 55]. In response, viruses often inhibit the formation of *bona fide* SGs (reviewed in [55]). Poliovirus 3C cleaves G3BP [56], whereas others, including alphaviruses, induce viral granules that sequester G3BP and/or other SG factors to favour viral replication [33, 34, 57–59]. Flavivirus and hepatitis C virus infections also bind SG components to increase their replication efficiency [58, 60] and G3BP1/2 are required for efficient CHIKV minus strand RNA transcription [48]. Together with our *in vivo* data on the proviral role of Rin in mosquitoes, this strongly suggests that viruses, including CHIKV, can utilize SG components (e.g. G3BP/Rin) for efficient replication in their natural hosts and/or vectors.

1. CHIKV nsP3 co-localizes with G3BP homologue Rasputin in cytoplasmic granules.
2. Two TFGD repeats within the C-term hypervariable domain of nsP3 interact with the NTF2-like domain of Rasputin.
3. Rasputin silencing does not disrupt the granular localization of nsP3 and has no profound effects on viral replication *in vitro.*
4. Rasputin silencing *in vivo* significantly reduces the CHIKV infection rate and dissemination in live *Aedes albopictus* mosquitoes.
